# A blunt traumatic giant pseudoaneurysm of the brachiocephalic artery: A case report from Somalia

**DOI:** 10.1016/j.ijscr.2024.109329

**Published:** 2024-02-02

**Authors:** Abdijalil Abdullahi Ali, Abdinafic Mohamud Hussein, Erkan Albay, Ali Abdulkadir Ali Siyad, Mohamed Omar Hassan, Said Abdirahman Ahmed

**Affiliations:** a Department of cardiovascular surgery at Mogadishu Somali Turkish Training and Research Hospital, Mogadishu, Somalia; b Department of Cardiology at Mogadishu Somali Turkish Training and Research Hospital, Mogadishu, Somalia

**Keywords:** Traumatic, Blunt, Brachiocephalic artery, Pseudoaneurysm

## Abstract

**Introduction:**

Pseudoaneurysm of the brachiocephalic artery is a rare condition that can occur as a result of various causes, including trauma, iatrogenic injury, and infection. The clinical presentation of brachiocephalic artery pseudoaneurysms can vary depending on the size and location of the pseudoaneurysm. The treatment options for innominate artery pseudoaneurysms include both surgical and endovascular approaches. Our goal of the study is to increase awareness and early detection of blunt injuries in the chest, clavicle, or sternoclavicular joint that may cause a vascular injury.

**Case presentation:**

We present here **A 24-year-old male came to present with an acute onset of dyspnea, stridor (**an abnormal, high-pitched respiratory sound produced by irregular airflow in a narrowed airway)**, a worsening cough, and chest pain that had been worsening over several months. His medical history was significant for blunt chest trauma secondary to a bicycle fall 3 months earlier.**

**Discussion:**

A traumatic giant pseudoaneurysm of the innominate artery is a rare but potentially life-threatening condition. Treatment options for brachiocephalic artery pseudoaneurysm include both endovascular and surgical approaches. This case report contributes to the current literature when any patient has a blunt injury in the chest, clavicle, or sternoclavicular joint and is highly suspect of a vascular injury. To increase awareness, we first need to exclude if there is any vascular injury, which helps to detect it early and intervene.

**Conclusion:**

Brachiocephalic artery traumatic large pseudoaneurysm is an uncommon but potentially fatal disorder that can arise from a number of different sources. Achieving favorable results requires prompt diagnosis and proper care, which may include open surgical repair and endovascular procedures. To better comprehend the condition and optimize its management approaches, more investigation and case studies are required.

## Highlights

• Pseudoaneurysm of the brachiocephalic artery is a rare condition that can occur as a result of various causes, including trauma, iatrogenic injury, and infection.
• Symptoms can range from mild to severe and potentially life-threatening, including stridor, tracheal compression, and respiratory distress.
• CTA is considered the most accurate diagnostic tool for identifying pseudoaneurysms in the brachiocephalic artery.
• The treatment options for innominate artery pseudoaneurysms include both surgical and endovascular approaches.

## 1 Introduction

Pseudoaneurysm of the brachiocephalic artery is a rare condition that can occur as a result of various causes, including trauma, iatrogenic injury, and infection [1,2].

The clinical presentation of brachiocephalic artery pseudoaneurysms can vary depending on the size and location of the pseudoaneurysm. Symptoms can range from mild to severe and potentially life-threatening, including stridor, tracheal compression, and respiratory distress [1,5,6]. The treatment options for innominate artery pseudoaneurysms include both surgical and endovascular approaches. Surgical repair may involve techniques such as lateral arteriography, patch angioplasty, primary end-to-end anastomosis, or placement of a prosthetic graft [14].

## 2 Case presentation

**Fig. 1:**
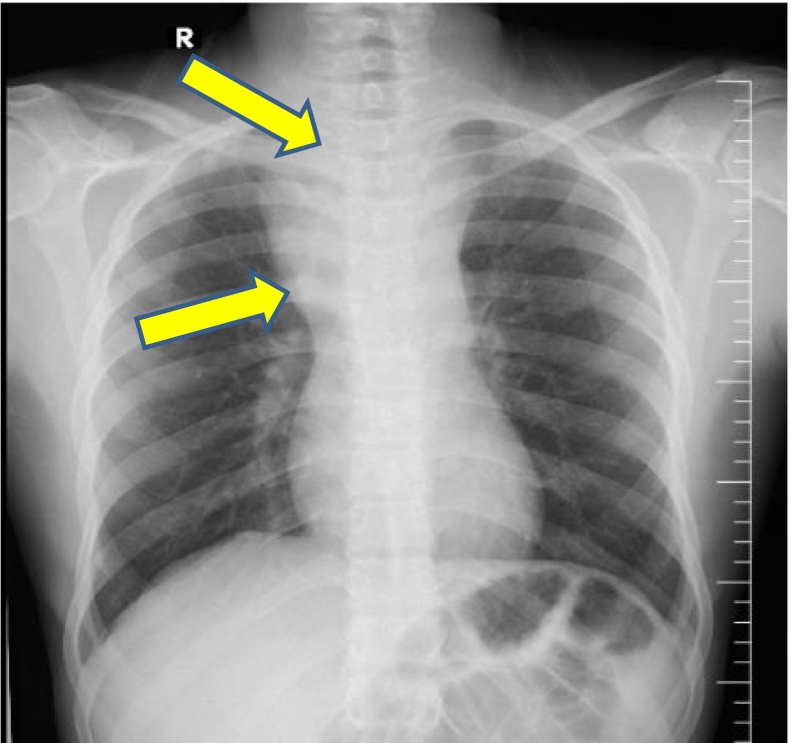
A chest x-ray showed a mediastinal mass and comminuted fracture of the sternoclavicular joint.

**Fig. 2 & 3:**
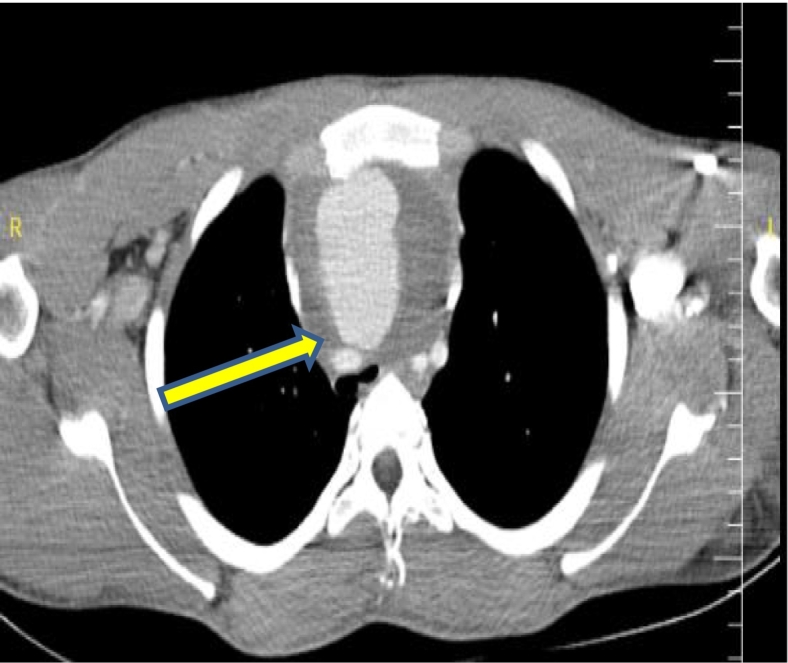

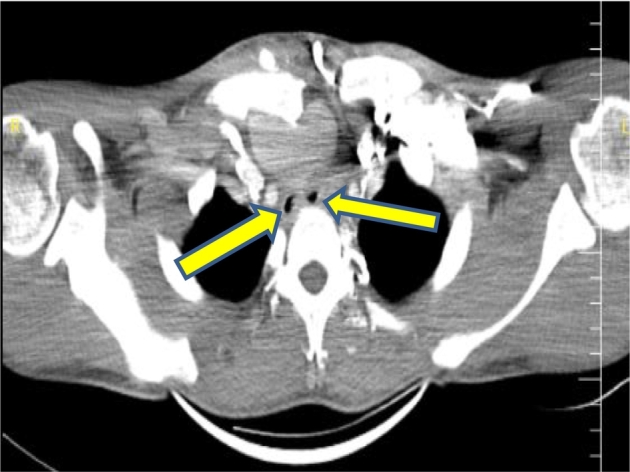
Computed tomography angiography with reconstruction revealed a pseudo-aneurysm of the brcahiocephalic artery and severe compresion in the main trachea.

A 24-year-old male came to the thoracic surgery clinic with an acute onset of dyspnea, stridor, a worsening cough, and chest pain that had been worsening over several months. A chest x-ray showed a mediastinal mass and comminuted fracture of the sternoclavicular joint (Fig. 1), and a subsequent computed tomography angiography with reconstruction revealed 5.3 cm of non-ruptured pseudo-aneurysm near the root innominate artery. (Fig. 2) and severe compresion in the trachea (Fig. 3). Thoracic surgery clinics refer patients to cardiovascular surgery clinics. His medical history was significant for blunt chest trauma secondary to a bicycle fall 3 months earlier. To alleviate compressive symptoms of the trachea and the absence of endovascular repair in our country, the patient was admitted to the ER and underwent open repair of his brachiocephalic pseudoaneurysm.

**Fig. 4:**
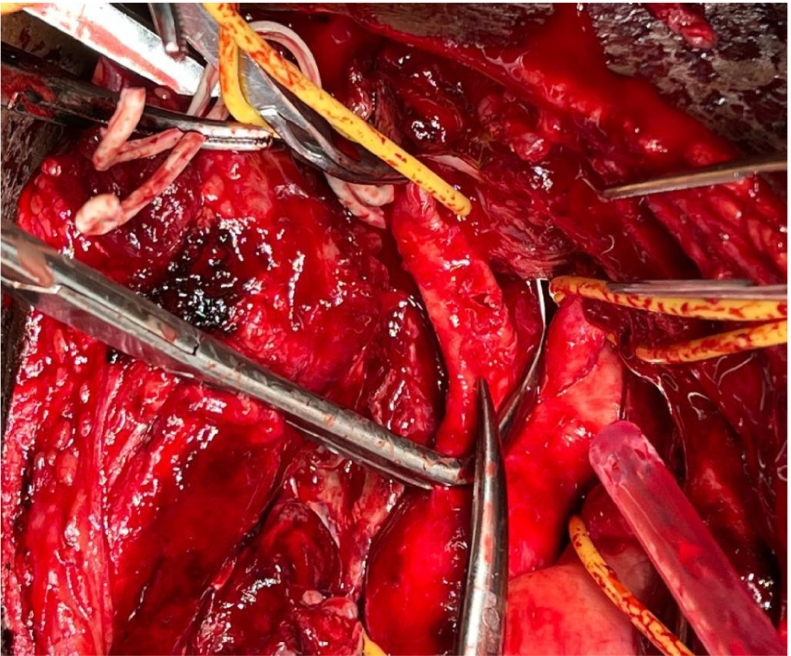
Intraoperatively, the site of the brachiocephalic injury.

**Fig. 5:**
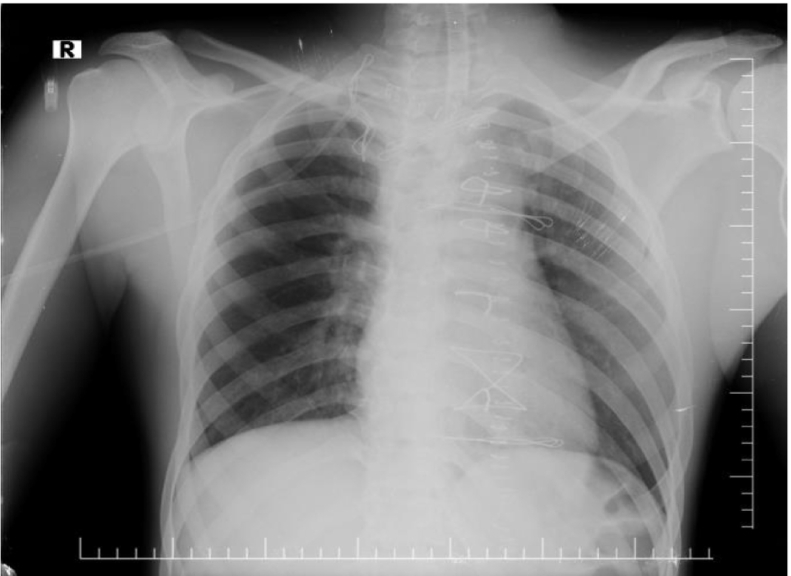
Normal post-op chest x-ray.

Surgical approach through the median sternotomy: after opening the sternum, an aneurysm sac ruptured and started bleeding controlled by digital pressure to avoid blind clamp occlusion. Fortunately, we controlled side clamps in the proximal brachiocephic artery, distal right sublavian, and common carotid arteries. The injury site (Fig. 4) was repaired with primary repair by using 5/0 prolen. One drain was inserted in the mediastinum, a normal post-op chest x-ray (Fig. 5). There were no complications from the surgery.

**Fig. 6:**
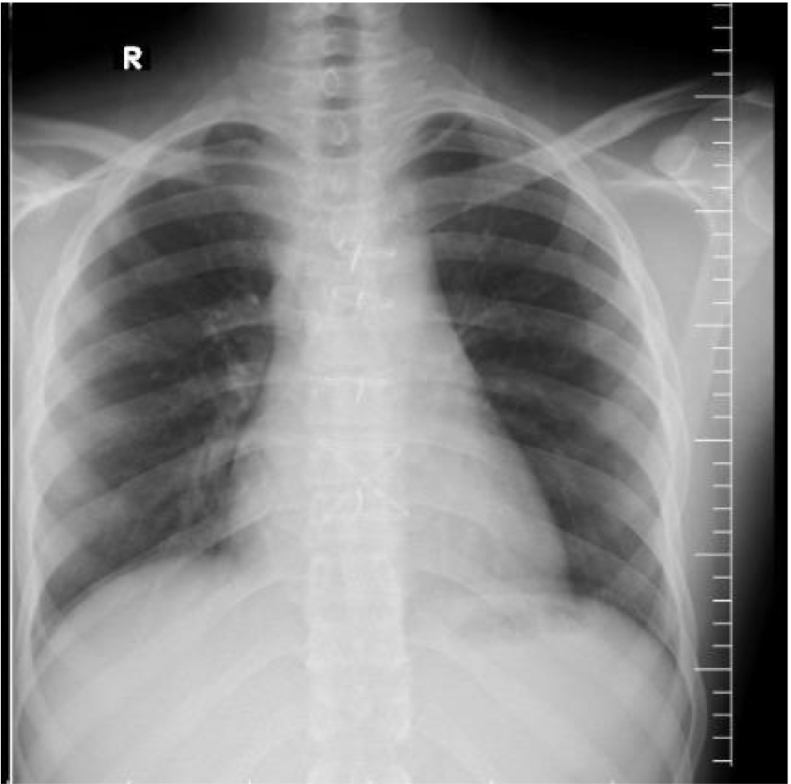
After 1 year of chest x-ray control.

The patient was discharged on the seventh day. Almost a year after the injury, the patient is doing well and has a normal chest x-ray (Fig. 6).

## 3 Discussion

A traumatic giant pseudoaneurysm of the innominate artery is a rare but potentially life-threatening condition [11]. Pseudoaneurysms of the innominate artery account for approximately 4 % of all innominate artery aneurysms [12]. Traumatic injuries, such as those from chest trauma or diagnostic procedures like mediastinoscopy, are the most common cause of brachiocephalic artery pseudoaneurysm [[1], [2], [3]]. Other causes include iatrogenic injury, such as during percutaneous coronary intervention (PCI) or central venous catheterization [2,4].

Infections, including infective endocarditis, have also been reported as causes of innominate artery pseudoaneurysm [13,15].

In some cases, pseudoaneurysms may be asymptomatic and discovered incidentally [7].

The diagnosis of brachiocephalic artery pseudoaneurysm is typically made using imaging modalities such as computed tomography angiography (CTA) or digital subtraction angiography (DSA). CTA is considered the most accurate diagnostic tool for identifying pseudoaneurysms in the brachiocephalic artery [1]. Rupture of the pseudoaneurysm can result in life-threatening hemorrhage [13]. Treatment options for brachiocephalic artery pseudoaneurysm include both endovascular and surgical approaches. Endovascular techniques, such as stent graft placement, have been used successfully to exclude the pseudoaneurysm and restore normal blood flow [[8], [9], [10]]. Surgical repair may be necessary in cases where endovascular techniques are not feasible or have failed [3,6].

A comparison of open vs. endovascular approaches based on a literature review to elaborate on endovascular techniques, such as stent-graft repair and coil embolization, has emerged as effective treatment options for innominate artery pseudoaneurysms.

These minimally invasive procedures offer the advantages of reduced morbidity and mortality compared to open surgical repair.

In cases where endovascular treatment is not feasible or contraindicated, open surgical repair, including interposition grafting or bypass grafting, may be considered [16,17].

This case report contributes to the current literature when any patient has a blunt injury in the chest, clavicle, or sternoclavicular joint and is highly suspect of a vascular injury. To increase awareness, we first need to exclude if there is any vascular injury, which helps to detect it early and intervene. This case has been reported in line with the Scare 2023 guideline [18].

## 4 Conclusion

Brachiocephalic artery traumatic large pseudoaneurysm is an uncommon but potentially fatal disorder that can arise from a number of different sources. Achieving favorable results requires prompt diagnosis and proper care, which may include open surgical repair and endovascular procedures. To better comprehend the condition and optimize its management approaches, more investigation and case studies are required.

## Ethical approval

According to our hospital rule, Ethical approval is only required in articles but not case reports.

## Funding

There is no funding source for this study.

## Author contribution

All authors contributed toward writing, analysis, drafting, and revising the paper and they gave final approval of the version to be published, and agree to be accountable for all aspects of the work.

## Guarantor

Abdijalil Abdullahi Ali

## Research registration number

1. Name of the registry: Abdijalil Abdullahi Ali.

2. Unique Identifying number or registration ID: Not applicable.

3. Hyperlink to your specific registration (must be publicly accessible and will be checked): Not applicable.

## Conflict of interest statement

I declare that there is no competing interest related to the study, authors, other individuals, or organizations.
